# Differential Gene Expression in Response to *Papaya ringspot virus* Infection in *Cucumis metuliferus* Using cDNA- Amplified Fragment Length Polymorphism Analysis

**DOI:** 10.1371/journal.pone.0068749

**Published:** 2013-07-09

**Authors:** Yu-Tsung Lin, Fuh-Jyh Jan, Chia-Wei Lin, Chien-Hung Chung, Jo-Chu Chen, Shy-Dong Yeh, Hsin-Mei Ku

**Affiliations:** 1 Department of Agronomy, National Chung Hsing University, Taichung, Taiwan; 2 Department of Plant Pathology, National Chung Hsing University, Taichung, Taiwan; New Mexico State University, United States of America

## Abstract

A better understanding of virus resistance mechanisms can offer more effective strategies to control virus diseases. *Papaya ringspot virus* (PRSV), *Potyviridae,* causes severe economical losses in papaya and cucurbit production worldwide. However, no resistance gene against PRSV has been identified to date. This study aimed to identify candidate PRSV resistance genes using cDNA-AFLP analysis and offered an open architecture and transcriptomic method to study those transcripts differentially expressed after virus inoculation. The whole genome expression profile of *Cucumis metuliferus* inoculated with PRSV was generated using cDNA-amplified fragment length polymorphism (cDNA-AFLP) method. Transcript derived fragments (TDFs) identified from the resistant line PI 292190 may represent genes involved in the mechanism of PRSV resistance. *C. metuliferus* susceptible Acc. 2459 and resistant PI 292190 lines were inoculated with PRSV and subsequently total RNA was isolated for cDNA-AFLP analysis. More than 400 TDFs were expressed specifically in resistant line PI 292190. A total of 116 TDFs were cloned and their expression patterns and putative functions in the PRSV-resistance mechanism were further characterized. Subsequently, 28 out of 116 candidates which showed two-fold higher expression levels in resistant PI 292190 than those in susceptible Acc. 2459 after virus inoculation were selected from the reverse northern blot and bioinformatic analysis. Furthermore, the time point expression profiles of these candidates by northern blot analysis suggested that they might play roles in resistance against PRSV and could potentially provide valuable information for controlling PRSV disease in the future.

## Introduction

Viral resistance is always a top priority to plant breeders. Numerous innate defense systems against pathogens have evolved in plants. For example, the cell wall and waxy cuticle of leaves and stems provide protection against physical invasion by insects such as aphids and whiteflies which are intermediate hosts for many plant viruses. In some plant species, the hypersensitive response (HR) is induced in the infected region of a leaf and restricts the spread of pathogens [1]. In addition, a substantial number of secondary metabolites such as salicylic acid (SA) or reactive oxygen species (ROSs) e.g., superoxide radical (O2^−^), hydrogen peroxide (H_2_O_2_) and hydroxyl radical (OH) are able to be produced to trigger the whole plant systemic acquired resistance (SAR). These signals can activate specific or nonspecific defense responses which contribute to a plant’s ability to protect itself against future pathogen infection [2].

The mechanism of viral resistance in plant is not fully understood due to the complicated nature of plant-virus interactions and the fact that only a few viral resistance genes have been identified so far [3], [4]. One hypothesis, gene for gene model, is based on the interactions between a plant resistance protein (R) and a pathogen avirulence protein (Avr) [5]–[7]. For example, direct interactions between *Tobacco mosaic virus* (TMV) replicase and tobacco N protein [8] or *Potato virus X* (PVX) coat protein and Rx1 or Rx2 of *Solanum tuberosum* [9] trigger defense response in tobacco and potato. A second hypothesis is that a virulence protein can disrupt the conformation of a guarded protein (guardee) which is guarded by R proteins [7], [10]. The change of conformation activates the R protein which then induces a signaling transduction pathway culminating in the resistance responses. The interaction of *Arabidopsis* resistance protein HRT, the *Turnip crinkle virus* capsid protein and their guardee protein TCV-interacting protein (TIP) is additional evidence supporting the guard hypothesis for virus-plant interactions [11]. However, 28 plant viral resistance genes have been identified from diverse plant species (including tobacco, *Arabidopsis*, potato, tomato and soybean [3], [4]), this “guard hypothesis” does not apply to most viral *Avr*- *R* gene pairs examined so far. Instead, it has been primarily the resistance mechanism against bacteria and fungi.

*Papaya ringspot virus* (PRSV), a member of the genus *Potyvirus* of the family *Potyviridae*, accounts for severe economical losses in papaya and cucurbit worldwide. The virus is transmitted by aphids, *Myzus persicae* or *Aphis gossypii,* in a nonpersistent manner in the field and is also spread by mechanical inoculation. Hallmark symptoms of PRSV in papaya include mosaic and leaf chlorosis, water-soaked streaking on the petiole and upper part of trunks, and the distortion of infected young leaves. The genetic organization of PRSV is similar to that of other *Potyvirus*, a positive single-stranded RNA that comprises poly-A tract and translates into a polyprotein. The polyprotein is then cleaved into the following mature proteins: VPg, P1, HC-Pro, P3, CI, 6K, NIa, NIb and CP [12]. Control of PRSV diseases in papaya has been focused on developing tolerant or resistant varieties; these varieties are rarely planted because of poor fruit quality and vigor [13], [14]. The cross protection strategy of inoculating papaya with a mild strain of PRSV (HA 5-1 or HA 6-1) provides resistance against severe PRSV strain infection in Taiwan [15], [16]. However, strain specificity, the technical difficulties associated with propagating pure strains of mild form of the virus and the unavailability of such mild strains limit the benefit of the approach [16]. An alternative strategy using RNA-mediated gene silencing [17] with transgenic plants expressing viral genes has been developed. Although this approach has been succeeded, resistance levels differ with environmental factors and plant development stages. Broad spectrum resistance against different PRSV isolates depends on the homology of transgenes with viral target genes [18]. Because the genetic divergence of different PRSV strains is correlated with their geographical distribution [19], transgenic lines against different viral strains must be developed individually for various papaya growing regions. For long-term protection of crops, developing novel resistant lines is generally considered the best strategy for the efficient control of viral diseases in papaya [20].

PRSV also causes tremendously losses in cucurbit crops. Interestingly, a PRSV resistance line of a wild cucurbit species called horned cucumber or jelly melon (*Cucumis metuliferus*) has been reported. This line, PI 292190, harboring a single dominant resistance gene, *Wmv*, showed resistance against PRSV and has been used for selecting attenuated strains of PRSV [16], [21], [22]. Alternatively, line Acc. 2459 is highly susceptible to PRSV and usually used as a regular virus propagation host. In this study, a comparative analysis of transcriptional profiles of *C. metuliferus* in response to PRSV infection is described. Currently, several approaches such as proteomics, cDNA microarray, suppression subtractive hybridization (SSH), and cDNA-AFLP are available for transcriptome analysis. cDNA-AFLP, a robust and high-throughput profiling tool for analyzing changes in mRNA level, was chosen for its high sensitivity, low labor cost, and ease of implementation when genome sequence information is not available [23]. An additional advantage of cDNA-AFLP is that its high sensitivity makes it possible to detect and identify rare transcripts [24]. Genes involved in virus resistance pathway and plant broader defenses are the focus of this study. Thus, aspects of the PRSV-induced *C. metuliferus* defense network have been revealed and putative functions involved in the disease resistance pathway have been identified. This is the first time several putative defense-related genes against PRSV have been characterized using cDNA-AFLP analysis in *C. metuliferus*.

## Results

### The Differential Responses of Susceptible *C. metuliferus* line Acc. 2459 and Resistant Line PI 292190 Against PRSV

**Figure 1:**
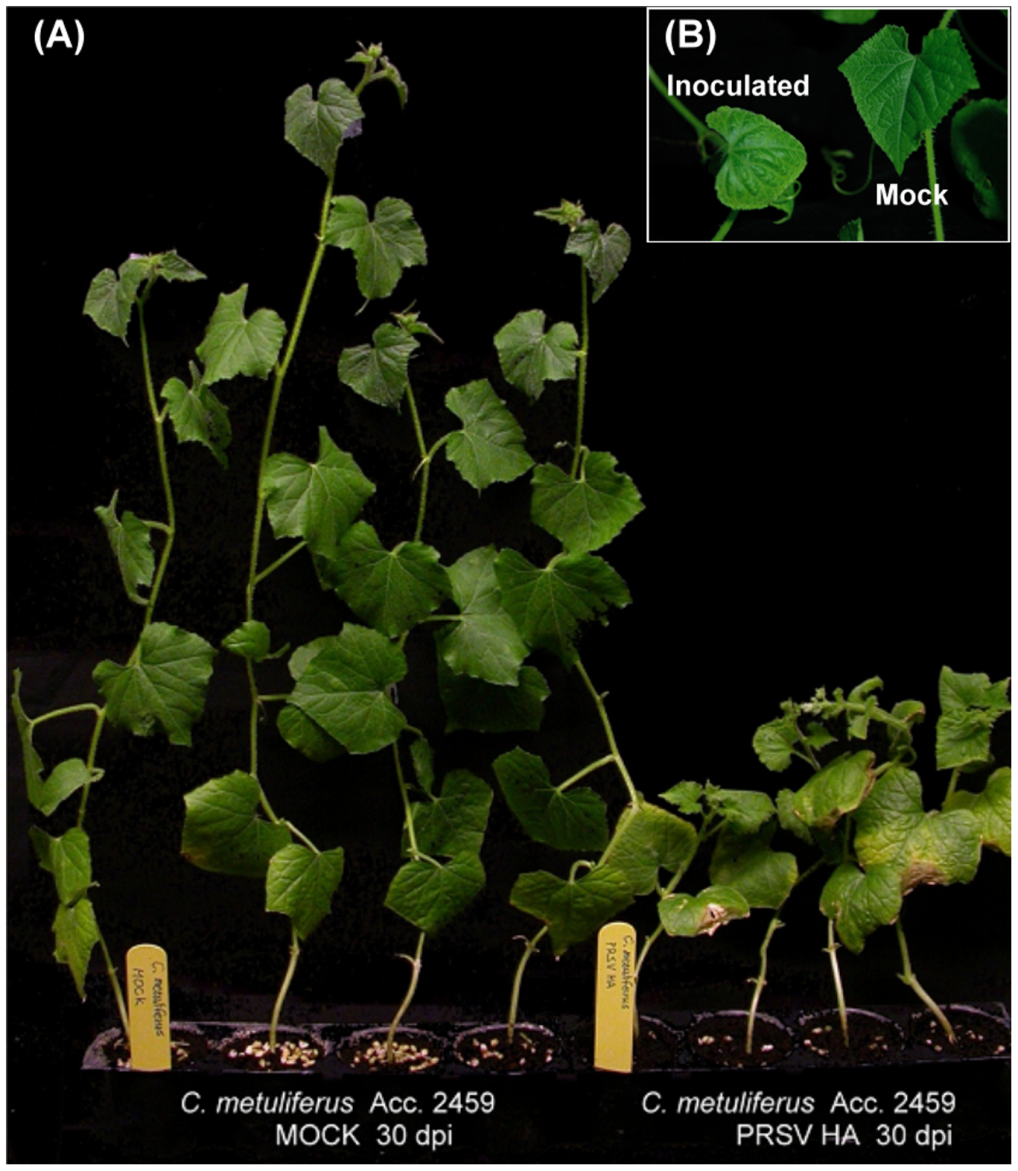
Symptoms on *C. metuliferus* susceptible line Acc. 2459 inoculated with PRSV. (A) *C. metuliferus* showing severe stunting, leaf distortion, and narrow leaf blades at 30 dpi. (B) Plants showed severe mosaic symptom and malformation in systemic leaves after 10 dpi.

PRSV infected *C. metuliferus* susceptible line Acc. 2459 showed severe symptoms and developmental defects at 7 to 10 days post-inoculation (dpi). The vegetative tissue exhibited stunting, malformation (Figure 1A, right panel), narrow leaf blades patterns on leaves (Figure 1B). No symptoms were observed in PRSV-inoculated resistant line PI 292190 indicating that resistance due to an extreme resistance or immune response but not HR. Differentially displayed cDNA libraries from susceptible line Acc. 2459 and resistant line PI 292190 were developed for cDNA-AFLP analysis. To ensure that differentially displayed genes in the inoculated resistant line PI 292190 were induced by virus infection rather than mechanical inoculation, the virion vigor of the inocula was determined. Because no symptoms were seen in the inoculated resistance line PI 292190 plants, the only way to evaluate infection ability was to apply the inoculum in susceptible line Acc. 2459 plants. Thus, after the inoculation of resistant line PI 292190, the same inoculum was immediately used for the inoculation of individuals of susceptible line Acc. 2459. Symptoms of these inoculated Acc. 2459 plants were observed to be the same as those on other Acc. 2459 plants inoculated with PRSV. This provided evidence that although no obvious response was seen in PI 292190 plants after virus infection, the plants has been exposed to a potent inoculum and therefore the changed gene expression of PRSV-inoculated resistant line PI 292190 was indeed caused by the PRSV infection.

### PRSV Proliferation and Movement in *C. metuliferus* line Acc. 2459

**Figure 2:**
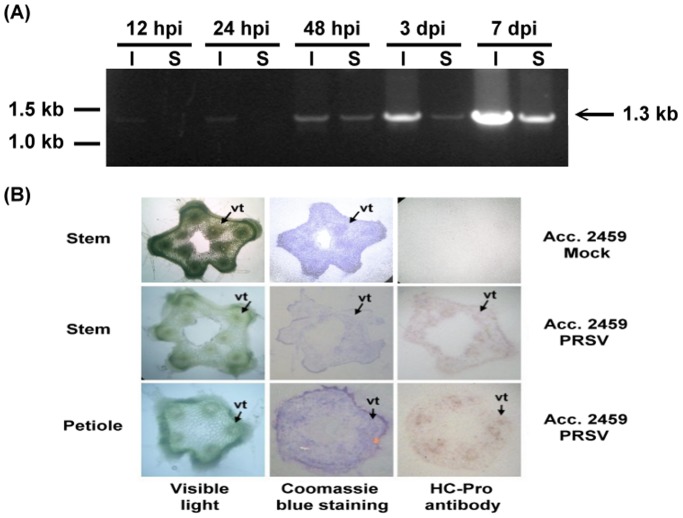
The detection of PRSV movement in *C. metuliferus* using RT-PCR and tissue printing. (A) RT-PCR analysis of HC-Pro gene (1.3 kb) in *C. metuliferus* susceptible line Acc. 2459 inoculated with PRSV. RNA samples were isolated from inoculated leaves (I) and systemic leaves (S) at five different time lines (12, 24, 48hpi, 3 and 7 dpi). (B) Tissue printing assay of HC-Pro protein accumulation in petiole and stem of susceptible line Acc. 2459 inoculated with PRSV at 7 dpi. The HC-Pro can be detected in the vascular tissues (column 3) of stem and petiole after immunostaining with anti-HC-Pro monoclonal antibodies (1∶5000 dilution) followed by goat anti-mouse alkaline phosphatase-conjugated secondary antibody (1∶5000 dilution). Color was developed with BCIP and NBT.

In this study, time lines of PRSV proliferation and movement in *C. metuliferus* susceptible line Acc. 2459 were investigated which provided the expression patterns of potential defense genes. Therefore, the systemic leaves from virus inoculated *C. metuliferus* susceptible line Acc. 2459 were excised from plants at different time lines and evaluated for virus proliferation and movement using RT-PCR. As shown in Figure 2A, PRSV was detected in all inoculated leaves at 12, 24 (weak RT-PCR bands), 48hours post-inoculation (hpi) and 3, 7 dpi. PRSV was detected in all systemic leaves with the exception of 12 and 24 hpi. In addition, tissue printing showed the PRSV HC-Pro protein in the stem and petiole at 7 dpi (Figure 2B). The results suggested that the proliferation and the movement of PRSV from inoculated leaves to systemic leaves in *C. metuliferus* susceptible line Acc. 2459 might occur during 24 and 48 hpi.

### cDNA-AFLP Analysis

**Figure 3:**
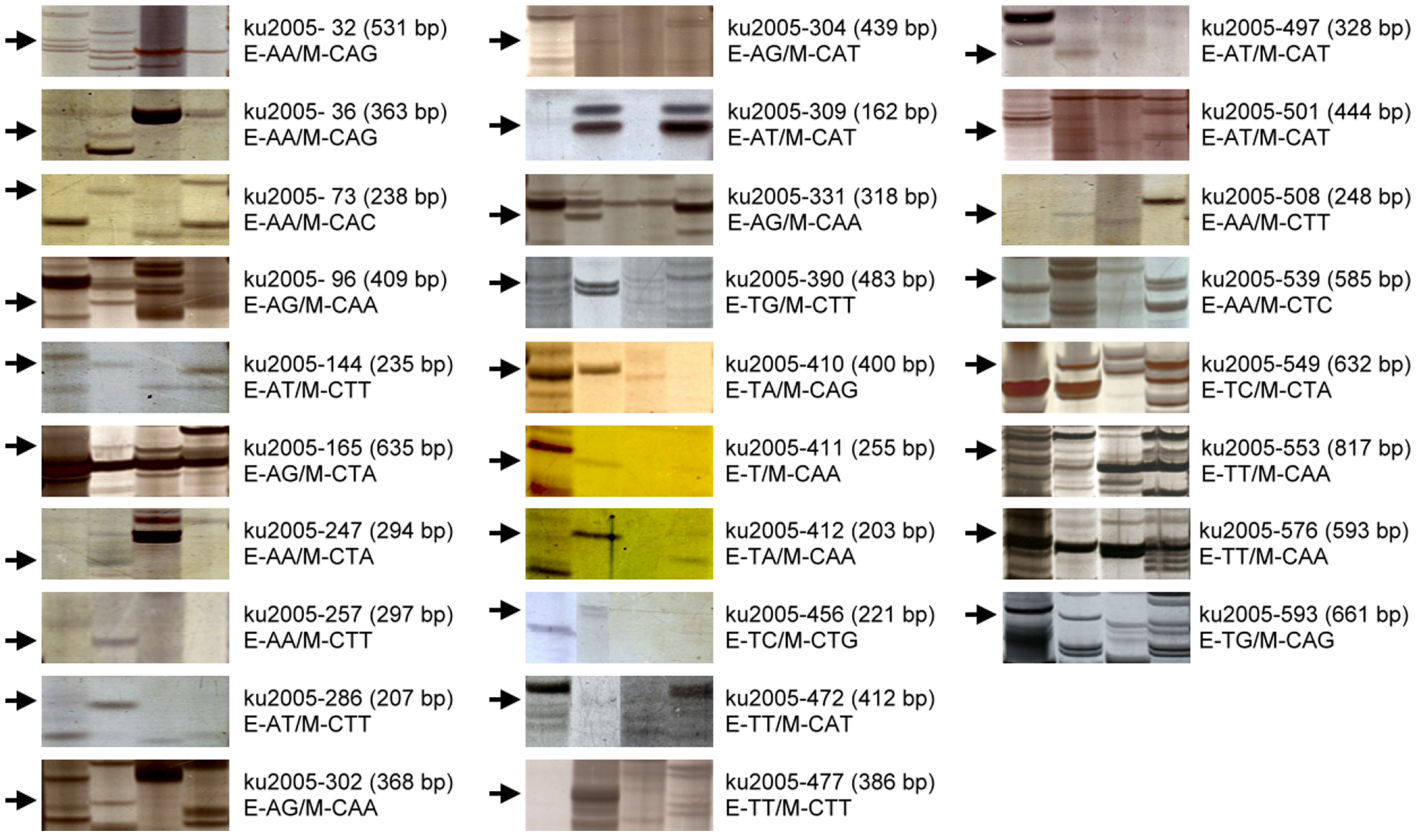
Differential display of cDNA-AFLP analysis for *C. metuliferus* responsive to PRSV and mock inoculation. Twenty-eight cDNA-AFLP fragments amplified from different primer combinations (E−/M−) were chosen to show the polymorphism between *C. metuliferus* inoculated with virus or sodium phosphate buffer (mock). The order of each panel from left to right is *C. metuliferus* susceptible line Acc. 2459-PRSV inoculated, resistant line PI 292190-PRSV inoculated, susceptible line Acc. 2459-Mock and resistant line PI 292190-Mock, respectively. The arrowhead represents the polymorphic fragments which were specifically induced in resistant line PI 292190-PRSV.

Bands showing polymorphic between Acc. 2459-PRSV and Acc. 2459-Mock or PI 292190-PRSV and PI 292190-Mock were identified in mixed RNA isolated at different times post inoculation using cDNA-AFLP. The time lines used in this study ranged from 24 hpi to 14 dpi and including stages prior to observable symptoms became noticeable (24, 48, 72, 96 hpi) and stages when symptoms were evident (7 and 14 dpi) in susceptible line Acc. 2459. Sixty four primer combinations were used in cDNA-AFLP analysis, and the molecular size of amplified fragments ranged from 50 to 3,000 bp on the 4% denaturing polyacrylamide sequencing gel. A total of 3,259 fragments was visualized in susceptible line Acc. 2459 inoculated with PRSV (Acc. 2459-PRSV), and this number was higher than those shown in susceptible line Acc. 2459-Mock (2,184), resistant line PI 292190-PRSV (1,865) and resistant line PI 292190-Mock (2,349). On average, 51 clear and unambiguous bands were obtained in susceptible line Acc. 2459-PRSV, 35 bands in susceptible line Acc. 2459-Mock, 30 bands in resistant line PI 292190-PRSV, and 37 bands in resistant line PI 292190-Mock. This indicated that the infection with PRSV resulted in the more widespread modulation of steady state mRNA level in susceptible line Acc. 2459-PRSV than in inoculated resistant line PI 292190-PRSV. More than 400 TDFs were specifically induced in resistant line PI 292190-PRSV and 267 in susceptible line Acc. 2459-PRSV. The polymorphic fragments induced specifically in resistant line PI 292190-PRSV (Figure 3) were excised from the gel, re-amplified and cloned onto a vector for sequencing. In all, 116 TDFs were obtained that gave rise to a selected differentially displayed library and sequence data. Although the cDNA fragments were produced by the digestion of *EcoR* I and *Mse* I restriction enzymes simultaneously and three combinations of restriction sites on the opposite end of fragments could be theoretically detected (*EcoR* I-*Mse* I, *EcoR* I-*EcoR* I and *Mse* I-*Mse* I), respectively, no cDNA fragments containing restriction sites of *EcoR* I-*EcoR* I were found. A total of 66 *Mse* I-*Mse* I and 50 *EcoR* I-*Mse* I fragments were produced in this study. In addition, the phenomenon of different cDNA-AFLP fragments representing the same candidate gene was also seen in this study. For example, the TDFs ku2005-36 (396 bp) and ku2005-512 (280 bp) both encoded a putative NtEIG-A1 protein but were identified from different selective primer combination of E-AA/M-CAG and E-AA/M-CTA, respectively (Table 1 and Table S1). Because the same gene was identified from different length cDNA restriction fragments, cDNA-AFLP seems to be suitable for detecting the expression of differentially displayed genes of interest.

**Table 1:** The putative functions (as determined by BLAST in GenBank) of the transcript derived fragments (TDFs) responsive to the resistance of *Cucumis metuliferus* infected with PRSV.

TDF	Size(bp)	I/C^a^	Accession number	Functional annotation	BLAST *E*-value	Restrictionsite^c^
Ku2005-32	531	I	MU46389	Serine/threonine kinase	5E-33	E/M
Ku2005-36	363	I	MU46830	NtEIG-A1; Early nodulin 16 precursor	1E-105	E/M
Ku2005-73	238	I	MU54221	DEAD-box RNA helicase	3E-12	M/M
Ku2005-96	409	C	MU48031	Leucine-rich repeat receptor-like kinase 1	1E-7	M/M
Ku2005-144	235	I	AAO42093	Putative cytochrome p450	2E-17	E/M
Ku2005-165	635	I	MU60832	Poly-galacturonate 4-α-galacturonosyl transferase	0	M/M
Ku2005-247	294	C	MU56499	Disease resistance protein (TIR-NBS-LRR)	2E-4	M/M
Ku2005-257	297	I	MU62576	Stellacyanin; cupredoxin	9E-57	M/M
Ku2005-286	207	I	MU49579	Dicarboxylate/tricarboxylate carrier	1E-101	E/M
Ku2005-302	368	I	MU47524	Thioredoxin h	1E-139	M/M
Ku2005-309	162	C	MU65146	Pathogen-responsive α-dioxygenase	2E-34	E/M
Ku2005-331	318	I	MU47481	Ran; small GTP-binding protein	1E-59	M/M
Ku2005-390	483	I	MU45662	NtEIG-E80; photoassimilate-responsive protein	0	M/M
Ku2005-410	400	I	MU50205	Hypothetical protein	1E-104	E/M
Ku2005-411	255	I	MU49201	Putative vesicle-associated membrane protein	1E-101	E/M
Ku2005-412	203	I	MU57990	Proteinase inhibitor	2E-53	E/M
Ku2005-456	221	I	CU115151	GA-like protein	1E-24	E/M
Ku2005-477	386	I	MU49841	Function unknown protein	1E-159	M/M
Ku2005-497	328	I	MU65146	Putative pathogen responsive α-dioxygenase	2E-57	E/M
Ku2005-501	444	C	MU45023	Function unknown protein	1E-15	M/M
Ku2005-508	248	I	MU47706	Putative mitochondrial ATP synthase	6E-85	E/M
Ku2005-539	585	I	XP483742	Protein L-isoaspartate-O-methyltransferase	1E-29	M/M
Ku2005-549	632	C	MU45188	Putative xyloglucan endotransglycosylase	0	M/M
Ku2005-553	817	C	MU46729	ckl3 (Casein kinase I-like 3)	0	M/M
Ku2005-576	593	I	MU50434	Aldose 1-epimerase	0	M/M
Ku2005-593	661	I	MU43624	Heat shock domain containing protein	7E-87	M/M
Ku2005-472	412	C	NS^b^			M/M
Ku2005-304	439	I	NS^b^			M/M

a The transcription of TDFs is induced by virus infection (I) or constitutive (C) in *C. metuliferus*.

b NS, No significant match.

c The TDFs contain either restriction enzyme site of *EcoR* I or *Mse* I at the both ends.

### Reverse Northern Blot and Northern Blot Analysis

**Figure 4:**
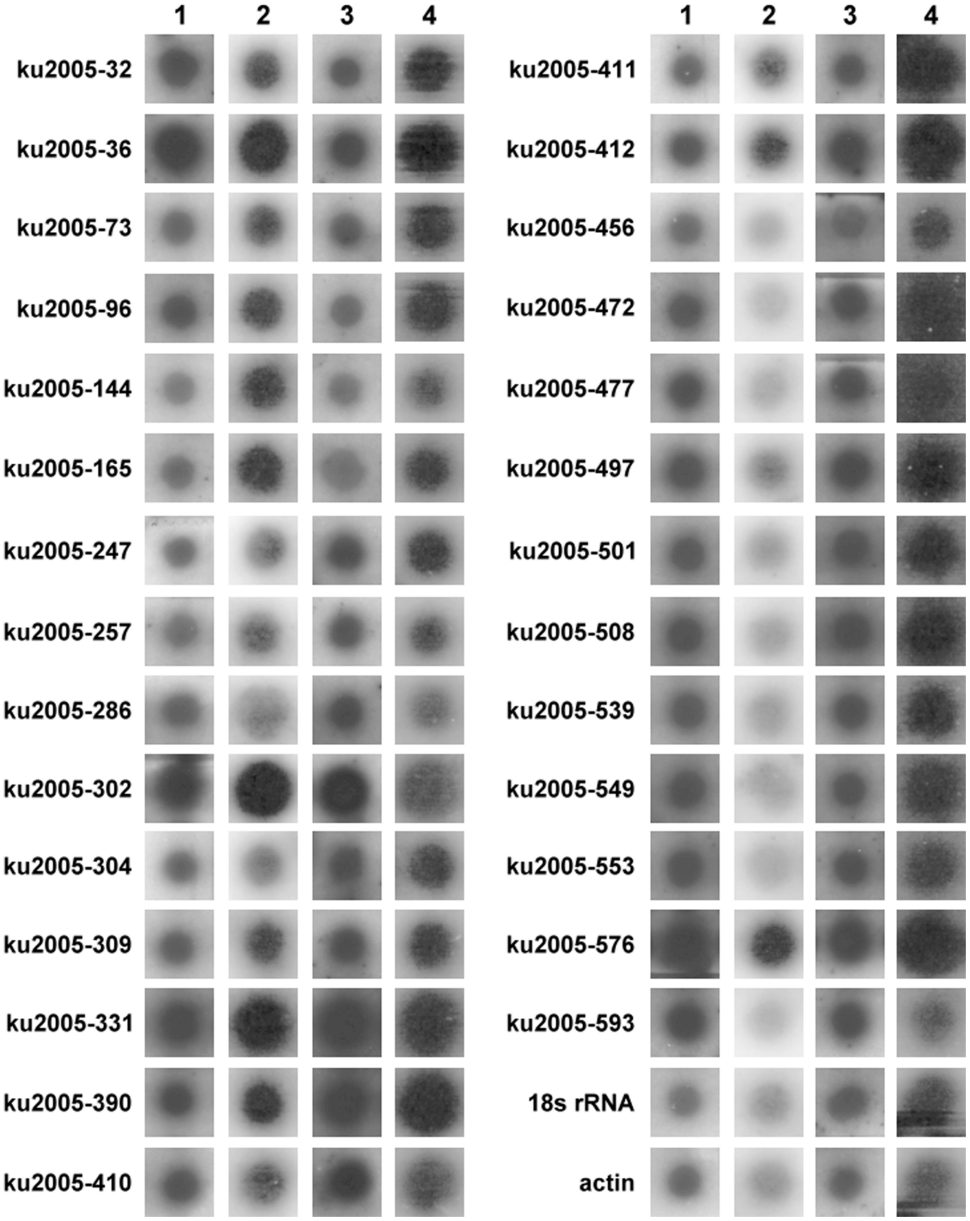
Reverse northern analysis of TDFs identified from *C. metuliferus* inoculated with PRSV or sodium phosphate buffer (mock). Reverse transcripted RNA mixture of *C. metuliferus* susceptible line Acc. 2459-PRSV inoculated (1), resistant line PI 292190-PRSV inoculated (2), susceptible line Acc. 2459-Mock (3) and resistant line PI 292190-Mock (4). The expression of 18S rRNA and actin isolated from *C. metuliferus* resistant line PI 292190 was used for normalization.

To confirm that candidates identified through cDNA-AFLP analysis were specifically related to PRSV resistance and to narrow down the candidate pool, reverse northern blot analysis was performed. Four identical membranes containing 116 candidate genes and internal control genes, actin and 18S ribosomal genes, were prepared and hybridized with isotope-labeled cDNAs made from susceptible line Acc. 2459-Mock or -PRSV and resistant line PI 292190-Mock or -PRSV. Most cDNA clones showed signal in both susceptible line Acc. 2459 and resistant line PI 292190 whether the plants were inoculated with PRSV or mock inoculated. The induced genes were defined as increasing expression by two fold in the resistant line PI 292190-PRSV as compared to resistant line PI 292190-Mock or in the susceptible line Acc. 2459-PRSV as compared to susceptible line Acc. 2459-Mock. Consequently, a total of 28 candidates showed increased expression level in resistant line PI 292190-PRSV, particularly candidates ku2005-36, 144, 165, 302, 331, 390, 412 and 576 which showed more than two-fold increased expression level in resistant line PI 292190-PRSV (Figure 4).

**Figure 5:**
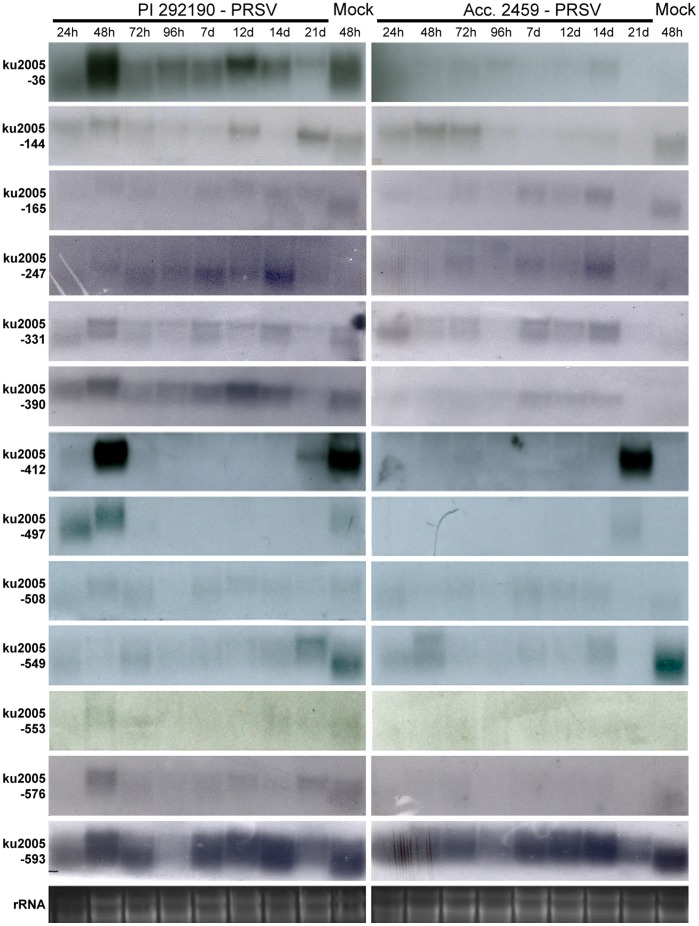
Candidate gene expression profile in *C. metuliferus* resistant line PI 292190 (left panel) and susceptible line Acc. 2459 (right panel) inoculated with PRSV or mock over eight time points post inoculation. The expression level of mock in two *C. metuliferus* lines is shown in the inoculation at 48 hpi. Ethidium bromide-stained rRNA is shown as loading control.

To further evaluate the expression of these 28 candidate genes, northern blot analysis was carried out to detect the transcripts at different time lines post inoculation. As shown in Figure 5, the transcripts of the candidates responding to different time points were detected in resistant line PI 292190 and susceptible line Acc. 2459. The transcription of candidates ku2005-36, 247, 390, 412, 497, 553 and 576 were induced at a much higher level in resistant line PI 292190-PRSV than in susceptible line Acc. 2459-PRSV. The highest expression level of these candidate RNAs in resistant line PI 292190-PRSV was shown at 48 hpi, except ku2005-247 for which the highest level was found at 14 dpi. The RNA levels of most induced candidates declined rapidly after 48 hpi except ku2005-36 and 412 which increased slightly at 12 and 21 dpi, respectively. No significant difference in transcription signals between susceptible line Acc. 2459 and resistant line PI 292190 was found in other candidates including ku2005-144, 165, 331, 508, 549 and 593. In addition, ku2005-412 showed a similar transcriptional level in resistant line PI 292190-PRSV (or -Mock) at 48 hpi and susceptible line Acc. 2459-PRSV at 21 dpi. Expression of ku2005-553 was seen only in resistant line PI 292190-PRSV (or -Mock); RNA from this candidate gene was not detected in susceptible line Acc. 2459.

### Functional Classification of *C. metuliferus* Line PI 292190 Genes Induced by PRSV Infection

**Figure 6:**
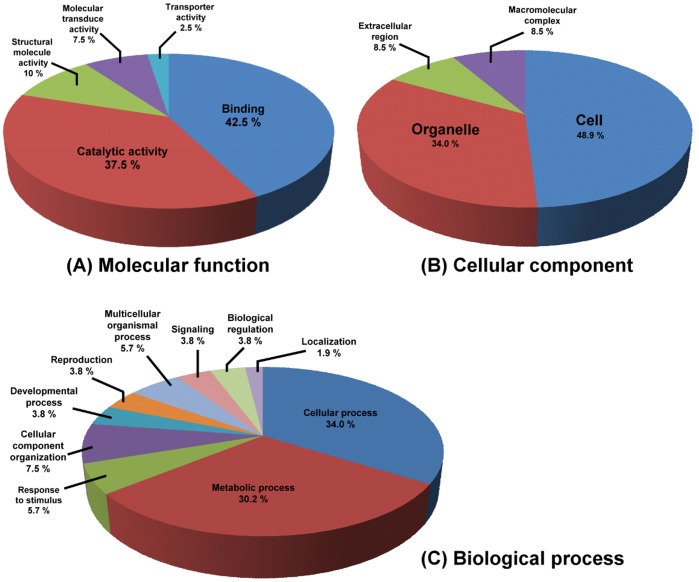
The pie charts of GO classification for cDNA-AFLP TDFs. GOslim classification of annotated cDNA-AFLP TDFs at level 2 for the three main GO vocabularies respectively: molecular function (A), cellular component (B) and biological process (C).

A total of 89 of 116 (76%) TDFs were found to have similarity to deposited genes, ESTs, or functional unknown proteins in GenBank using BLAST analysis (Table 1 and Table S1). Through gene ontology (GO) term mapping, 530 GO terms were retrieved from 89 TDFs by Blast2GO software according to the three main GO categories: molecular functions, cellular component and biological process (Figure 6). In the first category, molecular function, 42.5% and 37.5% of the TDFs showed putative binding and catalytic activity, respectively, and the remainder showed homology to proteins with structural molecular (10%), molecular transducer (7.5%) and transport (2.5%) activity (Figure 6A). Among the cellular components, most of TDFs were assigned in cell (48.9%) and organelle (34%) (Figure 6B). In addition, based on the third category, 34% and 30.2% of TDFs grouped by biological process have cellular and metabolic roles particularly in protein and carbohydrate metabolism. Other relevant TDFs categories (each accounting from 1.9−7.5%) included response to stimulus, cellular component organization, development, reproduction, multicellular organismal, signaling, biological regulation and localization process (Figure 6C). These results of GO term mapping suggest that the majority of TDFs involved in PRSV resistance operate to change the catalytic activity and metabolic process within cellular organelles. Among these TDFs, candidate “ku2005-247” had similarity to genes containing the NBS-LRR conserved protein domain which is a well-known domain of *R* gene.

## Discussion

### Differential Expression Analysis Using cDNA-AFLP Strategy

This is the first time PRSV resistance has been investigated in a non-model plant species, *C*. *metuliferus*. The cDNA-AFLP provided an open architecture and transcriptomic method for studying this wild species which led to the identification of candidate genes related to nonspecific (or basal) resistance in both susceptible line Acc. 2459-PRSV and resistant line PI 292190-PRSV (Figure S1). Because the survival of the obligate pathogens such as viruses lies on the nutrition provided from host plants, and virus infection makes plants changing gene expression patterns and the content of metabolisms to support virus proliferation. It has been suggested that gene expression of host plants infected with pathogens change predominantly in susceptible plants, especially during the late infection stage [25]–[27]. Indeed, the total number of polymorphic fragments observed in susceptible line Acc. 2459-PRSV (3259) was higher as compared to Acc. 2459-Mock (2184). In addition, a previous study reported that in potato, a greater number of differentially expressed genes are found in susceptible than in resistant cultivars in the later stage of *Potato virus Y* (PVY) infection [25]. Our results were in agreement with it when the total number of polymorphic fragments in susceptible line Acc. 2459-PRSV (3259) were compared to those in resistant line PI 292190-PRSV (1865). In summary, our studies provide the first large-scale cDNA-AFLP investigation of how genes in *C. metuliferus* respond to PRSV infection.

### Transition Expression of Candidate Genes for *C. metuliferus* Response to PRSV Inoculation

Because no comprehensive information about genes participating in virus-host interactions has been reported, it is challenging to decide which time lines (early or later stages) of expressed genes should be focused on. Only a few studies have shown the changes in gene expression that occur at different time lines after virus inoculation [25], [28]. Since no obvious symptom ever developed in *C. metuliferus* resistant line PI 292190 after PRSV-inoculation, the only clue that could confirm the success of virus inoculation was to inoculate with the same inoculums in the susceptible line Acc. 2459 which would display symptoms at 7 to 10 dpi. This suggested that the resistance mechanism might have been activated before the symptoms had a chance to develop [28]. In the current study, the proliferation and systemic movement of PRSV in the leaves of susceptible line Acc. 2459 were traced by RT-PCR analysis and also by tissue printing. By examining five time lines that spanned the period over which the onset of symptoms occurred, we were able to dissect virus proliferation and movement fully. These results indicated the systemic movement of PRSV in susceptible line Acc. 2459 occurred before 48 hpi.

Gene expression analyses conducted during the same time lines would reveal crucial information of host responses against virus infection. Therefore, 28 TDFs were evaluated for expression profile analysis using northern blot analysis. As shown in Figure 5, seven out of thirteen candidates were induced a much higher level in the resistant line PI292190-PRSV mostly at 48 hpi except candidate ku2005-247 responding at 14 dpi. Among these genes, the expression of ku2005-36 and ku2005-412 showed the highest accumulation in resistant line PI 292190-PRSV at 48 hpi and then decreased immediately. These results were similar to a report in tobacco that some defense-related genes are activated 48 h after challenging resistant plants with TMV in the resistant tobacco [28].

### The Putative Functions of TDF Candidates in Disease Resistance Responses

The candidate ku2005-73 showed sequence homology with melon DEAD-box ATP-dependent RNA helicase. The evidence of RNA helicase participating in response to biotic and abiotic stress emerged recently [29]–[31]. For example, DICER-LIKE 1 (DCL1), also a DEAD-box RNA helicase, is critical for miRNA biogenesis and RNA interference in *Arabidopsis* [32]. In addition, heterogeneous over-expression of rice OsBIRH1, a rice DEAD-box helicase, in transgenic *Arabidopsis* plants enhanced resistance to pathogen and oxidative stress [31]. Whether the induced expression of ku2005-73 in the resistant line PI 292190-PRSV is truly involved in the PRSV resistance mechanism remains an open question.

The candidate gene ku2005-412 encodes a protein with a putative function similar to protease inhibitors which were reported to be involved in plant reactions to stress and pathogen [33]–[35]. The expression peak of ku2005-412 in resistant line PI 292190 (48 hpi) is earlier and stronger than in susceptible line Acc. 2459 (21 dpi). The temporal and expression-level difference of candidate “ku2005-412” between two *C. metuliferus* varieties may implicate that PRSV resistance relies on deployment of defense genes at the earlier stage of PRSV infection. Plant protease inhibitors (PPIs) were grouped into at least ten families according to their sequences and structures [36]–[37] and the predicted product of ku2005-412 belongs to potato type I inhibitors family (InterPro accession no. IPR000864). This PPI family retains the specific effects towards chymotrypsin-like and elastase-like proteases. PPIs may also provide a new strategy for virus resistance as they inhibit the activity of virus proteases. To date, two *in vitro* experiments have shown that proteinase inhibitors, human cystatin C and corn cystatin II, have a slightly inhibitory effect on the activity of *Plum pox virus* HC-Pro protease [38]–[39]. Oryzacystatin I from rice conferred resistance against potyviruses when expressed in transgenic tobacco [40]. Although TMV does not use a polyprotein strategy for multiplication, an exogenous cockscomb cystatin (celostatin) prevents TMV-induced HR responses and represses TMV infection [33]. Two hypotheses for how PPIs confer viral resistance have been proposed. One suggests that PPIs interfere with proteolysis of viral proteases, the other suggests that other cellular components of the host essential for virus multiplication are affected. Evidence for the latter hypothesis is seen in transgenic tobacco expressing oryzacystatin I. Pleiotropic effects are observed in the cytosol of leaf cells and protein metabolism is altered [41]. In our study, the transgenic silencing of resistant line PI 292190 proteinase inhibitor gene has been conducted by RNAi approach and the transgenic plants show susceptibility to PRSV infection (unpublished data). The segregation of PRSV resistance and other morphological traits in the progeny (T_2_ generation) derived from these transgenic plants would elucidate more information about the role of ku2005-412 in the PRSV-resistance mechanism in resistant line PI 292190.

DNA sequence alignment of candidate ku2005-593 showed similarity with heat shock protein (HSP). It has been reported that HSP90 is required for activation of *Rx* resistance against PVX in *Nicotiana benthamiana* [42], [43]. HSP90, a molecular chaperone, can bind to SGT1 (suppressor of G2 allele of *skp*1) protein or other cofactors to form a complex that facilitates folding or stabilizes the Rx protein. In addition, the silencing of HSP90 compromises the resistance conferred by Rx and N proteins and subsequently infection of PVX and TMV increases [42], [44]. *P*. *syringae* resistance genes *RPM*1 and *RPS*2 which mediate resistances and cell death in *Arabidopsis* are also suppressed in HSP90 mutated lines or when HSP90 inhibitor are applied, respectively [45], [46]. It has been suggested that in the absence of HSP90, the misfolded proteins that result thereby interfering with the disease resistance responses.

### The Putative Signal Transduction Pathway Involved in *C. metuliferus* Resistance

After pathogen attack, susceptible and resistant host plants increase their respiration to support their survival [47]. Respiration consists of three main processes including glycolysis, tricarboxylic acid (TCA) cycle and electron transport. Among these, the product of glycolysis, acetyl-CoA, is carried into the mitochondria by mitochondrial carriers such as dicarboxylate-tricarboxylate carrier (DTC) [48] to yield ATP. In this study, the cDNA-AFLP candidate, ku2005-286, showed similarity to a melon unigene (MU49579) encoding a DTC protein. This carrier protein can transport a broad spectrum of dicarboxylates and tricarboxylates including phosphoenolpyruvate (PEP) which is the source for SA biosynthesis and also plays a vital role in SAR signal transduction [49].

The candidate, ku2005-497, putatively encodes an α-dioxygenase (α-DOX) which participates in plant fatty acid metabolism. The products of fatty acid metabolism include oxylipins such as jasmonic acid (JA) which is involved in stimulating biotic- and abiotic-stress responses [50]. Hamberg et al (2003) have found that the product of the α-DOX reaction can prevent the formation of necrotic lesions after infiltrating avirulent bacterial suspensions into tobacco leaves. This suggests that the activation of α-DOX is part of plant’s defense mechanisms and that this enzyme can protect plant tissues from programmed cell death [51].

The candidate ku2005-257 encodes a putative cupredoxin protein, one of blue copper-binding proteins including stellacyanins and plantacyanins which can bind a single copper atom. Cupredoxin can regulate Ca^2+^, Mg^2+^ or other ions in plants and participate in scavenging of ROSs which can trigger HR [52]. Plants have developed several approaches to precisely control ROSs to prevent their over-accumulation. It has been reported that the expression of cupredoxin can be induced by biotic- and abiotic-stress [53]. Therefore, the candidate gene ku2005-257 might be function in extreme resistance against PRSV seen in resistant line PI 292190.

The two candidates, ku2005-36 and 165, seem to be involved in cell wall cross-talk, another signal transduction pathway reported to influence plant defense, as the plant cell wall is the primary contact point during pathogen infection. Among these, candidate ku2005-36 is a nodule-specific plant-encoded protein (nodulin), a member of the cell wall membrane protein family. It has been reported that an *Arabidopsis* nodulin protein, AtGRP3, can bind to cell wall-associated receptor protein kinase (Wak1) to influence the oxidative status of cell wall proteins and play an important role in the defense mechanism. In addition, increased expression of *AtGRP3* enhanced *Pathogenesis Related 1* (*PR*-*1*) which is a molecular marker of SA-dependent defense protein [54]. Next, the candidate ku2005-165 shows similarity with genes encoding polygalacturonate 4-α-galacturonosyl transferase protein (PGA-GalAT; EC 2.4.1.43) which is involved in pectin biosynthesis in the plant cell wall. It is proposed that pectin structure controls pore size in the cell wall thereby restricting diffusion [55]. Young cell walls contain highly esterified homogalacturonan (HG) which is a linear chain of 1,4-linked α-D-galactopyranosyluronic acid (Gal*p*A) residues that does not gel readily. Contrarily, the rigidity of the wall in older cells of non-growing tissues and aphid-resistant plants is controlled by active pectin methylesterase (PME) through calcium-dependent esterizing. Some fungi or bacteria can produce pectin lyases that fragment cell wall pectins to generate carbon sources for themselves from Gal*p*A. Interestingly, Gal*p*A is also a signal molecule that was recognized as a trigger of host defense responses [56].

In addition to signal transduction pathway regulating plant resistance, the distribution of R proteins in the nucleus and cytoplasm also plays an important role in affecting the activity of R proteins and other defense-related proteins [57]. In this study, candidate ku2005-331 is predicted to be a plant Ran protein. Previous studies have shown that Ran proteins regulate the transport dynamics of molecules between nucleus and cytoplasm as well as microtubule organization during the cell cycle [58], [59]. For example, Ran proteins participating in nucleocytoplasmic regulation have been reported as a vital event to help a plant R protein, *Rx*, against PVX infection [60], [61]. As shown in Figure 5, expression of ku2005-331 was found in both line PI 292190 and Acc. 2459 inoculated with PRSV, implying that this gene could participate in the defense response in a non-specific way.

Reverse genetics provides a powerful tool for annotating the function of candidate genes. RNAi could be an efficient trigger for inducing gene silencing in plants [62], [63]. Thus molecular basis of PRSV resistance in *C. metuliferus* could be elucidated by silencing representatives of candidate genes isolated in this study. A transformation system for resistant line PI 292190 has been developed successfully in our lab [64] and should facilitate understanding the resistance mechanism of *C. metuliferus* against PRSV in the near future through the RNAi approach. Currently, we have obtained transgenic plants harboring RNAi construct of ku2005-412 that show the breaking down the immune response of resistant line PI 292190 successfully. This has provided a direct evidence of the potential function of ku2005-412 involving in PRSV resistance in *C. metuliferus* (Lin et al unpublished data).

### Conclusion

Little is known about the molecular interactions between PRSV and *C. metuliferus* line PI 292190 which shows extreme resistance to PRSV infection. The efficient and powerful strategy of cDNA-AFLP analysis combined reverse northern and time-point northern blot analysis provide us an opportunity to decipher this question. Comparing gene expression patterns through cDNA-AFLP analysis, more than 400 TDFs were found in the resistant line PI 292190-PRSV. Subsequently 116 of these TDFs were cloned successfully and sequential narrow down processes were conducted to identify a total of 28 candidate genes. A general correlation of expression pattern, sequence information and ontology analysis of these 116 TDFs have offered us insight into the *C. metuliferus* resistance mechanism. Among these candidate genes, candidate ku2005-412 and ku2005-247 were notable because their sequences are predicted to be homologous to a proteinase inhibitor protein and a NBS-LRR domain-containing resistance protein, respectively. Others including ku2005-36, 73, 165, 257, 286, 331, 497 and 593 might be involved in signal transduction pathways, ROSs scavenging, or the metabolisms of proteins or RNAs.

## Materials and Methods

### Plant Materials and Virus Inoculation

*C. metuliferus* susceptible line Acc. 2459 and resistant line PI 292190 seeds were germinated in compost and grown in a temperature-controlled greenhouse at 25–28°C. Virus inoculum source for subsequent mechanical inoculation was propagated in susceptible line Acc. 2459 infected with a PRSV Hawaiian isolate (PRSV-HA) at 7 dpi. The susceptible line Acc. 2459 and resistant line PI 292190 plants at the 4th or 5th true-leaf stage were used for time-point inoculation experiments with fresh inoculum sap diluted 1∶100 (w/v) in sodium phosphate buffer (0.01 M, pH 7.0). All inoculated plants were kept in the same greenhouse for further analysis. Parallel mock inoculations were also conducted with sodium phosphate buffer in both *C. metuliferus* lines. There were six different time points for sampling leaves from *C. metuliferus*: 24-, 48-, 72-, 96- hpi and 7, 14 dpi. A total of six plants were used for each time-point treatment and the plant leaves were collected, frozen immediately in liquid nitrogen, and stored at −80°C before extracting total RNA. The total RNA of six time points were sampled equally and mixed for constructing cDNA libraries. In total, four individual cDNA libraries (susceptible line Acc. 2459-PRSV, susceptible line Acc. 2459- Mock, resistant line PI 292190-PRSV, and resistant line PI292190-Mock) were constructed.

### Virus Detection Using Immunoblot Analysis and RT-PCR

To localize the virus in stem and petiole tissue, a tissue immunoblot analysis was performed with inoculum from susceptible line Acc. 2459 inoculated with PRSV at 7 dpi. Briefly, after inoculation of virus, the inoculated leaves were labeled and then excised from plants at 7 dpi. The stem and petiole of individual plants were cut across their axes using a double-edged razor blade and the cut surfaces were sandwiched between two nitrocellulose membranes 20s. Tissue prints were allowed to air dry and stored at 4°C before using. One of the membranes was stained with coomassie blue and the other one was immunostained with anti-HC-Pro monoclonal antibodies diluted 1∶5000 with TSW buffer (10 mM Tris, 0.9% NaCl, 0.25% gelatin, 0.1% Triton X-100 and 0.02% SDS) followed by goat anti-mouse immunoglobulin (1∶5000 dilution with TSW buffer). The membrane was then washed by adding alkaline phosphatase buffer and detected with 1-step BCIP/NBT system (Pierce, Rockford, IL). Furthermore, the inoculated and systemic leaves at five time lines (12, 24, 48 hpi, 3 and 7 dpi) were collected separately for RT-PCR analysis using HC-Pro specific primer pairs (forward: 5-AGAATGACGTGGCTGAAAAATTC-3; reverse: 5-CGCCGACAATGTAGTGCTTCAT-3).

### RNA Extraction and cDNA-AFLP Analysis

The extraction of total RNA was carried out using the methods described by Napoli and colleagues [65] except that the RNA pellet was resuspended in DEPC-treated water. The RNA quantity and quality were determined by using spectrophotometer and gel-electrophoresis. The Poly(A)+ RNA was purified from total RNA using PolyATract® mRNA isolation systems (Promega, Madison, WI, USA) according to the manufacturer’s instructions. The isolated mRNA was converted into double-stranded cDNA using the cDNA synthesis system with oligo-dT primer as per the manufacturer's protocol (Roche Applied Science). The concentration of synthesized cDNA was measured using a spectrophotometer.

cDNA-AFLP was conducted according to the method of Bachem and colleagues [66] with some modifications. A total of 500 ng of cDNA was digested simultaneously with *EcoR* I and *Mse* I (Fermentas, St. Leon-Rot, Germany), and the digested products were ligated to the *EcoR* I adapter and the *Mse* I adapter. The ligated products were diluted at ten fold with distilled water and subjected to two rounds of PCR amplification. The first-round PCR reactions (also called pre-amplification) were performed using the combination of *EcoR* I primer (5-GACTGCGTACCAATTC-3) and *Mse* I primer (5-GATGAGTCCTGAGTAAC-3) which contained the *Mse* I adapter sequence plus a selective nucleotide cytosine at 3′ end. The PCR profile was: 20 cycles at 94°C, 30 s; 56°C, 60 s; 72°C, 60 s. The first-round PCR products were diluted 50-fold with distilled water and used for secondary PCR reactions. A total of 64 primer combinations used for the subsequent selective amplification were *EcoR* I primer having two selective nucleotides at 3′ end: AA, AC, AG, AT, TA, TC, TG, TT and *Mse* I primer containing three selective nucleotides at 3′ end: CAA, CAC, CAG, CAT, CTA, CTC, CTG, CTT). The PCR profile was: 1 cycle at 94°C, 30 s; 65°C, 30 s; 72°C, 60 s and 13 cycles at 94°C, 30 s; 65°C, 30 s; 72°C, 60 s, followed by a 0.7°C decrease of the annealing temperature every cycle, and followed by 23 cycles at 94°C, 30 s; 56°C, 30 s; 72°C, 60 s. The PCR products of cDNA-AFLP analysis were resolved by electrophoresis in 4% denatured polyacrylamide sequencing gel and the banding patterns were obtained by silver staining described by Neilan and colleagues [67].

### Isolation and Cloning of Transcript-derived Fragment (TDF)

The polymorphic fragments which presented in PI 292190-PRSV or PI 292190-Mock but were absent in Acc. 2459-Mock and Acc. 2459-PRSV were excised from the gel using a sharp razor blade and DNA was isolated using crush and soak method [68]. The eluted products were re-amplified in a final volume of 25 µl using the pre-amplification primer of cDNA-AFLP analysis. To ensure precision and reproducibility, the reamplified products were verified in 4% denatured polyacrylamide sequencing gel and compared to the size of original corresponding cDNA-AFLP polymorphic fragments. Finally, these fragments were subsequently cloned into the yT&A vector (Yeastern Biotech Co., Ltd, Taipei, Taiwan) according to the manufacturer’s method.

### Sequence Analysis and Functional Classification of TDFs

TDFs were sequenced on an automated ABI Prism 377 sequencer (Applied Biosystems, Foster City, CA) at Genomics BioSci & Tech Co. (Taipei, Taiwan) using M13 promoter primer or M13 terminator primer. The vector sequences of TDFs were trimmed off and the resulting sequences were aligned to the Genbank database (http://www.ncbi.nlm.nih.gov/) and also to Cucurbit Genomics (CG) database (http://www.icugi.org/) by using BLASTN and BLASTX algorithms. Subsequently, these candidate gene sequences were filtered by blasting in melon unigene and the ID numbers of melon unigenes were recorded to classify these TDFs. In summary, TDFs were grouped into three main categories based on reports of scientific literatures, their predicted functions, biological processes, and cellular components using categorizing tool in CG Database.

The GO annotation analysis was achieved by means of Blast2GO software [69] and the TDFs were classified according to their role of cellular components, biological processes and molecular functions in plant, respectively. The results of Blast2GO were exported in a text format and exchanged to generate pie charts using Microsoft Excel spread sheets.

### Reverse Northern Blot and Northern Blot Analysis

For reverse northern blot analysis, equal amounts of each clone DNA (10 µg) which contained the TDFs were denatured by the sodium hydroxyl (NaOH) method and transferred directly on to a pre-wetted nylon membrane (Perkin Elmer Life Science, Boston, MA) using a dot-blot apparatus (Bio-Rad Laboratories, Richmond, CA). The membrane was baked dry at 80°C for two hours to immobilize the DNA. The probes were made from the total RNA derived from the mixtures of six time-points of susceptible line Acc. 2459 or resistant line PI 292190 inoculated with PRSV HA or mock treatment and subsequently reverse transcribed into double stranded cDNA using the oligo-dT primer. The doubled-stranded cDNA probes were radioactively labeled by the random hexamer primer method [70] in the presence of ^32^P-dATP. Non-incorporated nucleotides were removed using a Sephadex G-50 column. After heat denaturing, aliquots of the isotope labeled cDNAs were added to each membrane for hybridization at 60°C. The membranes were washed three times for 10 min with washing buffer (0.1% SDS, 2X SSC) at 60°C. The expression signal of each spots was evaluated by ScanAlyze software. For analyzing each spot, the intensity of each spot was conducted to normalize by subtracting background intensity of *C. metuliferus* housekeeping genes, including the actin and 18S ribosomal genes. After normalization, the signal of each spot was compared among the different treatments. The differentially expressed candidate genes identified from the reverse northern blot analysis were further confirmed by conventional northern blot. A total of 10 µg RNA aliquots isolated from susceptible line Acc. 2459-PRSV, resistant line PI 292190-PRAV at different time points (24, 48, 72, 96 hpi and 7, 12, 14, 21 dpi) and from susceptible line Acc. 2459-Mock, resistant line PI 292190-Mock at 48 hpi were separated by electrophoresis in a denaturing 1% agarose gel containing 5% MOPS. After separating, the RNA was blotted onto nylon membrane and the membrane was baked at 80°C for two hours to immobilize RNA. The method of hybridization and wash used for northern blot analysis were the same as performed for reverse northern blot.

## Supporting Information

Figure S1 **cDNA-AFLP profile of** ***C. metuliferus*** **susceptible line Acc. 2459 and resistant line PI 292190 inoculated with PRSV and sodium phosphate buffer (mock), respectively.** RNA sample was subjected to cDNA-AFLP analysis with different primer pairs (E−/M−). Panel 1: susceptible line Acc. 2459-PRSV; 2: resistant line PI 292190-PRSV; 3: susceptible line Acc. 2459-Mock; 4: resistant line PI 292190-Mock. The TDFs are marked with an arrowhead. The top part shows that the TDFs present in both susceptible line Acc. 2459-PRSV and resistant line PI 292190-PRSV but absent in mock treatment. These non-specific TDFs were proposed to be related to virus attack and played roles in basal resistance. The bottom part shows two specific TDFs specific in resistant line PI 292190: these TDFs potentially offer *C. metuliferus* resistance against to PRSV infection.
(PDF)

Table S1 **The putative functions (as determined by BLAST in GenBank) of the other transcript derived fragments (TDFs) responsive to the resistance of** ***C. metuliferus*** **infected with PRSV.**
(PDF)

## References

[1] Iakimova ET, Michalczuk L, Woltering EJ (2005) Hypersensitive cell death in plants: its mechanisms and role in plant defence against pathogens. J Fruit Ornam Plant Res 13: 135–158.

[2] Pieterse CMJ, Dicke M (2007) Plant interactions with microbes and insects: from molecular mechanisms to ecology. Trends Plant Sci 12: 564–569. 17997347 10.1016/j.tplants.2007.09.004

[3] Moffett P (2009) Mechanisms of recognition in dominant R gene mediated resistance. Adv Virus Res 75: 1–33. 20109662 10.1016/S0065-3527(09)07501-0

[4] Truniger V, Aranda MA (2009) Recessive resistance to plant viruses. Adv Virus Res 75: 119–159. 20109665 10.1016/S0065-3527(09)07504-6

[5] Flor HH (1956) The complementary genic systems in flax and flax rust. Adv Genet 8: 29–54.

[6] Hammond-Kosack KE, Jones JD (1996) Resistance gene-dependent plant defense responses. Plant Cell 8: 1773–1791. 8914325 10.1105/tpc.8.10.1773 PMC161314

[7] van der Biezen EA, Jones JD (1998) Plant disease-resistance proteins and the gene-for-gene concept. Trends Biochem Sci 23: 454–456. 9868361 10.1016/s0968-0004(98)01311-5

[8] Erickson FL, Holzberg S, Calderon-Urrea A, Handley V, Axtell M, et al (1999) The helicase domain of the TMV replicase proteins induces the N-mediated defence response in tobacco. Plant J 18: 67–75. 10341444 10.1046/j.1365-313x.1999.00426.x

[9] Bendahmane A, Kohn BA, Dedi C, Baulcombe DC (1995) The coat protein of *Potato virus X* is a strain-specific elicitor of *Rx*1-mediated virus resistance in potato. Plant J 8: 933–941. 8580963 10.1046/j.1365-313x.1995.8060933.x

[10] Dangl JL, Jones JD (2001) Plant pathogens and integrated defense responses to infection. Nature 411: 826–833. 11459065 10.1038/35081161

[11] Ren T, Qu F, Morris TJ (2000) HRT gene function requires interaction between a NAC protein and viral capsid protein to confer resistance to *Turnip crinkle virus* . Plant Cell 12: 1917–1926. 11041886 10.1105/tpc.12.10.1917 PMC149129

[12] Yeh SD, Jan FJ, Chiang CH, Doong TJ, Chen MC, et al (1992) Complete nucleotide sequence and genetic organization of *Papaya ringspot virus* RNA. J Gen Virol 73: 2531–2541. 1402799 10.1099/0022-1317-73-10-2531

[13] Conover RA, Litz RE, Malo SE (1986) ‘Cariflora’- a *Papaya ringspot virus*-tolerant papaya for South Florida and the Caribbean. HortScience 21: 1072.

[14] Dillon S, Ramage C, Ashmore S, Drew RA (2006) Development of a codominant CAPS marker linked to PRSV-P resistance in highland papaya. Theor Appl Genet 113: 1159–1169. 16932884 10.1007/s00122-006-0375-2

[15] Yeh SD, Gonsalves D, Wang HL, Namba R, Chiu RJ (1988) Control of *Papaya ringspot virus* by cross protection. Plant Dis 72: 375–380.

[16] Yeh SD, Cheng YH (1989) Use of resistant *Cucumis metuliferus* for selection of nitrous-acid induced attenuated strains of *Papaya ringspot virus* . Phytopathology 79: 1257–1261.

[17] Chiang CH, Wang JJ, Jan FJ, Yeh SD, Gonsalves D (2001) Comparative reactions of recombinant *Papaya ringspot viruses* with chimeric coat protein (CP) genes and wild-type viruses on CP-transgenic papaya. J Gen Virol 82: 2827–2836. 11602796 10.1099/0022-1317-82-11-2827

[18] Bau HJ, Cheng YH, Yu TA, Yang JS, Yeh SD (2003) Broad-spectrum resistance to different geographic strains of *Papaya ringspot virus* in coat protein gene transgenic papaya. Phytopathology 93: 112–120. 18944164 10.1094/PHYTO.2003.93.1.112

[19] Wang CH, Yeh SD (1997) Divergence and conservation of the genomic RNAs of Taiwan and Hawaii strains of papaya ringspot potyvirus. Arch Virol 142: 271–285. 9125043 10.1007/s007050050076

[20] Fermin GA, Castro LT, Tennant PF (2010) CP-transgenic and non-transgenic approaches for the control of papaya ringspot: current situation and challenges. Transgenic Plant J 4: 1–15.

[21] Provvidenti R, Robinson RW (1977) Inheritance of resistance to *Watermelon mosaic virus* 1 in *Cucumis metuliferus* . J Hered 68: 56–57.

[22] Provvidenti R, Gonsalves D (1982) Resistance to *Papaya ringspot virus* in *Cucumis metuliferus* and its relationship to resistance to *Watermelon mosaic virus* 1. J Hered 73: 239–240.

[23] Fukumura R, Takahashi H, Saito T, Tsutsumi Y, Fujimori A, et al (2003) A sensitive transcriptome analysis method that can detect unknown transcripts. Nucleic Acids Res 31: e94. 12907746 10.1093/nar/gng094 PMC169986

[24] Reijans M, Lascaris R, Groeneger AO, Wittenberg A, Wesselink E, et al (2003) Quantitative comparison of cDNA-AFLP, microarrays, and GeneChip expression data in *Saccharomyces cerevisiae* . Genomics 82: 606–618. 14611802 10.1016/s0888-7543(03)00179-4

[25] Baebler S, Krecic-Stres H, Rotter A, Kogovsek P, Cankar K, et al (2009) PVY^NTN^ elicits a diverse gene expression response in different potato genotypes in the first 12 h after inoculation. Mol Plant Pathol 10: 263–275. 19236574 10.1111/j.1364-3703.2008.00530.x PMC6640473

[26] Gyetvai G, Sonderkaer M, Gobel U, Basekow R, Ballvora A, et al (2012) The transcriptome of compatible and incompatible interactions of potato (*Solanum tuberosum*) with Phytophthora infestans revealed by DeepSAGE analysis. PLoS ONE 7: e31526. 22328937 10.1371/journal.pone.0031526 PMC3273468

[27] Tao Y, Xie Z, Chen W, Glazebrook J, Chang HS, et al (2003) Quantitative nature of *Arabidopsis* responses during compatible and incompatible interactions with the bacterial pathogen *Pseudomonas syringae* . Plant Cell 15: 317–330. 12566575 10.1105/tpc.007591 PMC141204

[28] Oh SK, Lee S, Chung E, Park JM, Yu SH, et al (2006) Insight into types I and II nonhost resistance using expression patterns of defense-related genes in tobacco. Planta 223: 1101–1107. 16482435 10.1007/s00425-006-0232-1

[29] Owttrim GW (2006) RNA helicases and abiotic stress. Nucleic Acids Res 34: 3220–3230. 16790567 10.1093/nar/gkl408 PMC1484253

[30] Vashisht AA, Tuteja N (2006) Stress responsive DEAD-box helicases: a new pathway to engineer plant stress tolerance. J Photochem Photobiol B 84: 150–160. 16624568 10.1016/j.jphotobiol.2006.02.010

[31] Li D, Liu H, Zhang H, Wang X, Song F (2008) OsBIRH1, a DEAD-box RAN helicase with functions in modulating defense responses against pathogen infection and oxidative stress. J Exp Bot 59: 2133–2146. 18441339 10.1093/jxb/ern072 PMC2413282

[32] Park W, Li J, Song R, Messing J, Chen X (2002) CARPEL FACTORY, a Dicer homolog, and HEN1, a novel protein, act in microRNA metabolism in *Arabidopsis thaliana* . Curr Biol 12: 1484–1495. 12225663 10.1016/s0960-9822(02)01017-5 PMC5137372

[33] Gholizadeh A, Santha IM, Kohnehrouz BB, Lodha ML, Kapoor HC (2005) Cystatins may confer viral resistance in plants by inhibition of a virus-induced cell death phenomenon in which cysteine proteinases are active: cloning and molecular characterization of a cDNA encoding cysteine-proteinase inhibitor (celostatin) from *Celosia cristata* (crested cock’s comb). Biotechnol Appl Biochem 42: 197–204. 15842197 10.1042/BA20050029

[34] Koiwa H, Bressan RA, Hasegawa PM (1997) Regulation of protease inhibitors and plant defense. Trends Plant Sci 2: 379–384.

[35] Zhang X, Liu S, Takano T (2008) Two cysteine proteinase inhibitors from *Arabidopsis thaliana*, AtCYSa and AtCYSb, increasing the salt, drought, oxidation and cold tolerance. Plant Mol Biol 68: 131–143. 18523728 10.1007/s11103-008-9357-x

[36] Habib H, Fazili KM (2007) Plant protease inhibitors: a defense strategy in plants. Biotech Mol Biol Rev 2: 68–85.

[37] De Leo F, Volpicella M, Licciulli F, Liuni S, Gallerani R, et al (2002) PLANT-PIs: a database for plant protease inhibitors and their genes. Nucleic Acids Res 30: 347–348. 11752333 10.1093/nar/30.1.347 PMC99076

[38] Garcia JA, Cervera MT, Riechmann JL, Lopez-Otin C (1993) Inhibitory effects of human cystatin C on Plum pox potyvirus proteases. Plant Mol Biol 22: 697–701. 8343605 10.1007/BF00047410 PMC7089253

[39] Wen R, Zhang SC, Michaud D, Sanfacon H (2004) Inhibitory effects of cystatins on proteolytic activities of the Plum pox potyvirus cysteine proteinases. Virus Res 105: 175–182. 15351491 10.1016/j.virusres.2004.05.008

[40] Gutierrez-Campos R, Torres-Acosta JA, Saucedo-Arias LJ, Gomez-Lim MA (1999) The use of cysteine proteinase inhibitors to engineer resistance against potyviruses in transgenic tobacco plants. Nat Biotechnol 17: 1223–1226. 10585723 10.1038/70781

[41] van der Vyver C, Schneidereit J, Driscoll S, Turner J, Kunert K, et al (2003) Oryzacystatin I expression in transformed tobacco produces a conditional growth phenotype and enhances chilling tolerance. Plant Biotechnol J 1: 101–112. 17147747 10.1046/j.1467-7652.2003.00010.x

[42] Lu R, Malcuit I, Moffett P, Ruiz MT, Peart J, et al (2003) High throughput virus-induced gene silencing implicates heat shock protein 90 in plant disease resistance. EMBO J 22: 5690–5699. 14592968 10.1093/emboj/cdg546 PMC275403

[43] Boter M, Amigues B, Peart J, Breuer C, Kadota Y, et al (2007) Structural and functional analysis of SGT1 reveals that its interaction with HSP90 is required for the accumulation of Rx, an R protein involved in plant immunity. Plant Cell 19: 3791–3804. 18032631 10.1105/tpc.107.050427 PMC2174866

[44] Liu Y, Burch-Smith T, Schiff M, Feng S, Dinesh-Kumar SP (2004) Molecular chaperone HSP90 associates with resistance protein N and its signaling proteins SGT1 and Rar1 to modulate an innate immune response in plants. J Biol Chem 279: 2101–2108. 14583611 10.1074/jbc.M310029200

[45] Hubert DA, Tornero P, Belkhadir Y, Krishna P, Takahashi A, et al (2003) Cytosolic HSP90 associates with and modulates the *Arabidopsis* RPM1 disease resistance protein. EMBO J 22(21): 5679–5689. 14592967 10.1093/emboj/cdg547 PMC275404

[46] Takahashi A, Casais C, Ichimura K, Shirasu K (2003) HSP90 interacts with RAR1 and SGT1 and is essential for RPS2-mediated disease resistance in *Arabidopsis* . Proc Natl Acad Sci USA 100: 11777–11782. 14504384 10.1073/pnas.2033934100 PMC208834

[47] Bolton MD (2009) Primary metabolism and plant defense-fuel for the fire. Mol Plant Microbe Interact 22: 487–497. 19348567 10.1094/MPMI-22-5-0487

[48] Picault N, Palmieri L, Pisano I, Hodges M, Palmieri F (2002) Identification of a novel transporter for dicarboxylates and tricarboxylates in plant mitochondria. Bacterial expression, reconstitution, functional characterization, and tissue distribution. J Biol Chem 277: 24204–24211. 11978797 10.1074/jbc.M202702200

[49] Chen Z, Zheng Z, Huang J, Lai Z, Fan B (2009) Biosynthesis of salicylic acid in plants. Plant Signal Behav 4: 493–496. 19816125 10.4161/psb.4.6.8392 PMC2688294

[50] Howe GA, Jander G (2008) Plant immunity to insect herbivores. Annu Rev Plant Biol 59: 41–66. 18031220 10.1146/annurev.arplant.59.032607.092825

[51] Hamberg M, Sanz A, Rodriguez MJ, Calvo AP, Castresana C (2003) Activation of the fatty acid α-dioxygenase pathway during bacterial infection of tobacco leaves. J Biol Chem 278: 51796–51805. 14522973 10.1074/jbc.M310514200

[52] Nersissian AM, Immoos C, Hill MG, Hart PJ, Williams G, et al (1998) Uclacyanins, stellacyanins, and plantacyanins are distinct subfamilies of phytocyanins: plant-specific mononuclear blue copper proteins. Protein Sci 7: 1915–1929. 9761472 10.1002/pro.5560070907 PMC2144163

[53] Jansen C, Korell M, Eckey C, Biedenkopf D, Kogel KH (2005) Idnetification and transcriptional analysis of powdery mildew-induced barley genes. Plant Sci 168: 373–380. 10.1007/s11103-004-0275-2 15604661

[54] Park AR, Cho SK, Yun UJ, Jin MY, Lee SH, et al (2001) Interaction of the Arabidopsis receptor protein kinase Wak1 with a glycine-rich protein, AtGRP-3. J Biol Chem 276: 26688–26693. 11335717 10.1074/jbc.M101283200

[55] Mohnen D (2008) Pectin structure and biosynthesis. Curr Opin Plant Biol 11: 266–277. 18486536 10.1016/j.pbi.2008.03.006

[56] D’Ovidio R, Mattei B, Roberti S, Bellincampi D (2004) Polygalacturonases, polygalacturonase-inhibiting proteins and pectic oligomers in plant-pathogen interactions. Biochim Biophys Acta 1696: 237–244. 14871664 10.1016/j.bbapap.2003.08.012

[57] Meier I, Somers D (2011) Regulation of nucleocytopalsmic trafficking in plants. Curr Opin Plant Biol 14: 538–546. 21764628 10.1016/j.pbi.2011.06.005

[58] Clarke PR, Zhang C (2001) Ran GTPases: a master regulator of nuclear structure and function during the eukaryotic cell division cycle? Trends Cell Biol 11: 366–371. 11514190 10.1016/s0962-8924(01)02071-2

[59] Hoelz A, Blobel G (2004) Cell biology: Popping out of the nucleus. Nature 432: 815–816. 15602540 10.1038/432815a

[60] Tameling WI, Baulcombe DC (2007) Physical association of the NB-LRR resistance protein Rx with a Ran GTPase-activating protein is required for extreme resistance to Potato virus X. Plant Cell. 19: 1682–1694. 10.1105/tpc.107.050880 PMC1913736 17526750

[61] Tameling WI, Nooijen C, Ludwig N, Boter M, Slootweg E, et al (2010) RanGAP2 mediates nucleocytoplasmic partitioning of the NB-LRR immune receptor Rx in the Solanaceae, thereby dictating Rx function. Plant Cell 22: 4176–4194. 21169509 10.1105/tpc.110.077461 PMC3027175

[62] Helliwell C, Waterhouse P (2003) Construsts and methods for high-throughput gene silencing in plants. Methods 30: 289–295. 12828942 10.1016/s1046-2023(03)00036-7

[63] Meister G, Tuschl T (2004) Mechanisms of gene silencing by double-stranded RNA. Nature 431: 343–349. 15372041 10.1038/nature02873

[64] Lin Y-T, Lin C-W, Chung C-H, Su M-H, Ho H-Y, et al (2011) In vitro regeneration and genetic transformation of Cucumis metuliferus through cotyledon organogenesis. HortScience 46: 616–621.

[65] Napoli C, Lemieux C, Jorgensen R (1990) Introduction of a chimeric chalcone synthase gene into petunia results in reversible co-suppression of homologous genes in trans. Plant Cell 2: 279–289. 12354959 10.1105/tpc.2.4.279 PMC159885

[66] Bachem CWB, Oomen RJFJ, Visser RGF (1998) Transcript imaging with cDNA-AFLP: a step-by-step protocol. Plant Mol Biol Rept 16: 157–173.

[67] Neilan BA, Leigh DA, Rapley E, McDonald BL (1994) Microsatellite genome screening: rapid non-denaturing, non-isotopic dinucleotide repeat analysis. Biotechniques 17: 708–712. 7833033

[68] Sambrook J, Russell DW (2006) Isolation of DNA fragments from polyacrylamide gels by the crush and soak method. NY, USA: Cold Spring Harbor Laboratory Press. 10.1101/pdb.prot100479 30710028

[69] Conesa A, Gotz S, Garcia-Gomez JM, Terol J, Talon M, et al (2005) Blast2GO: a universal tool for annotation, visualization and analysis in functional genomics research. Bioinformatics 21: 3674–3676. 16081474 10.1093/bioinformatics/bti610

[70] Feinberg AP, Vogelstein B (1983) A technique for radiolabeling DNA restriction endonuclease fragments to high specific activity. Anal Biochem 132: 6–13. 6312838 10.1016/0003-2697(83)90418-9

