# Supplementary material for: Differential Gene Expression in Response to Papaya ringspot virus Infection in Cucumis metuliferus Using cDNA- Amplified Fragment Length Polymorphism Analysis
Source: PLoS One. 2013 Jul 9;8(7):e68749. doi: 10.1371/journal.pone.0068749 (PMC3706314; doi:10.1371/journal.pone.0068749)
Supplement: Figure S1 — cDNA-AFLP profile of C. metuliferus susceptible line Acc. 2459 and resistant line PI 292190 inoculated with PRSV and sodium phosphate buffer (mock), respectively. RNA sample was subjected to cDNA-AFLP analysis with different primer pairs (E−/M−). Panel 1: susceptible line Acc. 2459-PRSV; 2: resistant line PI 292190-PRSV; 3: susceptible line Acc. 2459-Mock; 4: resistant line PI 292190-Mock. The TDFs are marked with an arrowhead. The top part shows that the TDFs present in both susceptible line Acc. 2459-PRSV and resistant line PI 292190-PRSV but absent in mock treatment. These non-specific TDFs were proposed to be related to virus attack and played roles in basal resistance. The bottom part shows two specific TDFs specific in resistant line PI 292190: these TDFs potentially offer C. metuliferus resistance against to PRSV infection. (PDF) [file pone.0068749.s001.pdf]

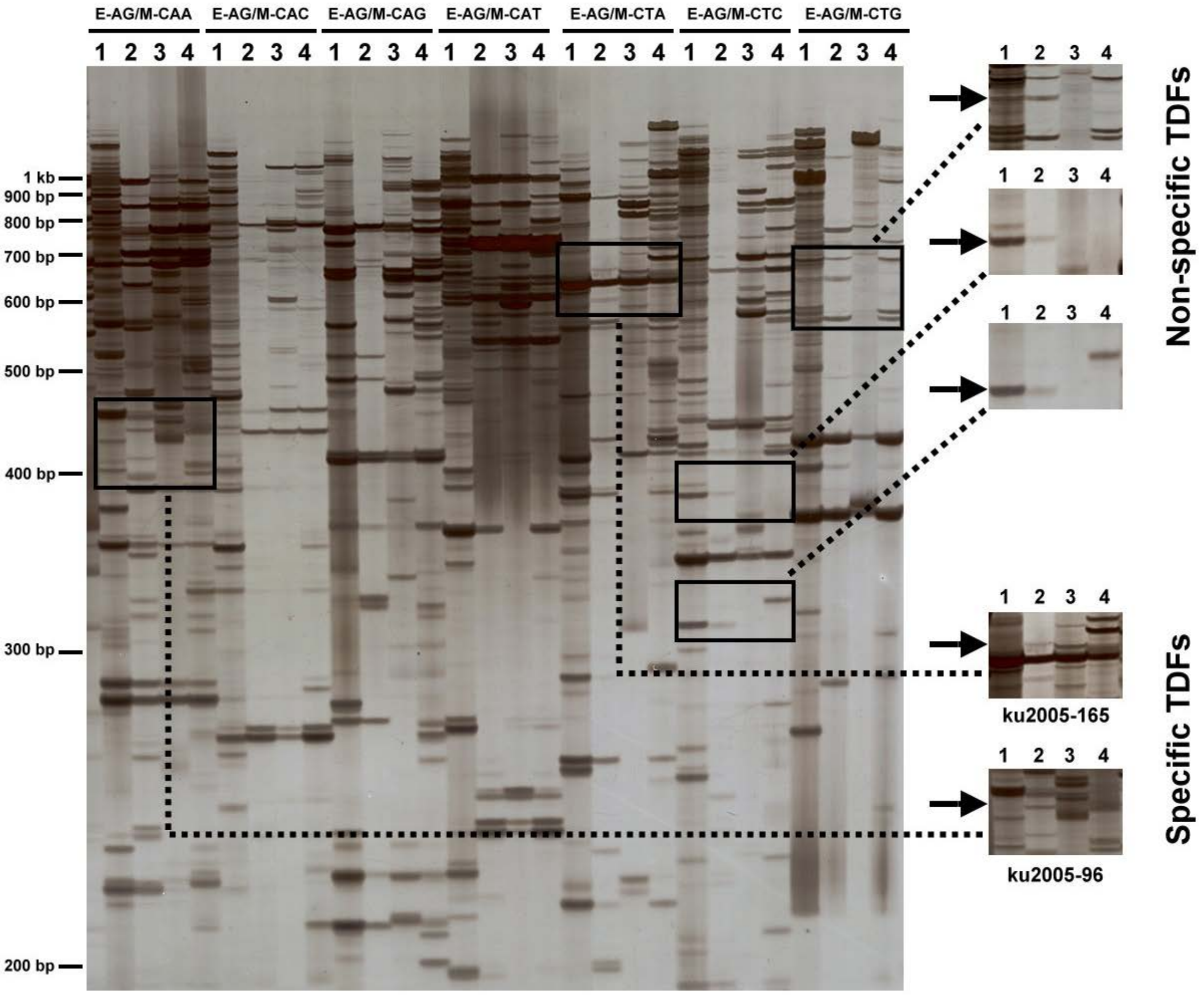

Figure S1. cDNA-AFLP profile of *C. metuliferus* susceptible line Acc. 2459 and resistant line PI 292190 inoculated with PRSV and sodium phosphate buffer (mock), respectively. RNA sample was subjected to cDNA-AFLP analysis with different primer pairs (E-/M-). Panel 1: susceptible line Acc. 2459-PRSV; 2: resistant line PI 292190-PRSV; 3: susceptible line Acc. 2459-Mock; 4: resistant line PI 292190-Mock. The TDFs are marked with an arrowhead. The top part shows that the TDFs present in both susceptible line Acc. 2459-PRSV and resistant line PI 292190-PRSV but absent in mock treatment. These non-specific TDFs were proposed to be related to virus attack and played roles in basal resistance. The bottom part shows two specific TDFs specific in resistant line PI 292190: these TDFs potentially offer *C. metuliferus* resistance against to PRSV infection.
