# Supplementary material for: Differential Gene Expression in Response to Papaya ringspot virus Infection in Cucumis metuliferus Using cDNA- Amplified Fragment Length Polymorphism Analysis
Source: PLoS One. 2013 Jul 9;8(7):e68749. doi: 10.1371/journal.pone.0068749 (PMC3706314; doi:10.1371/journal.pone.0068749)
Supplement: Table S1 — The putative functions (as determined by BLAST in GenBank) of the other transcript derived fragments (TDFs) responsive to the resistance of C. metuliferus infected with PRSV. (PDF) [file pone.0068749.s002.pdf]

Table S1. The putative functions (as determined by BLAST in GenBank) of the other transcript derived fragments (TDFs) responsive to the resistance of *C. metuliferus* infected with PRSV

TDF	Size (bp)	I/C[a]	Accession number	Functional annotation	BLAST *E*-value	Restriction site[b]
ku2005-11	291	I	BAD87519	putative Golgi autoantigen	2E-10	M+M
ku2005-22	565	I	NP565018	oxidoreductase	4E-48	M+M
ku2005-30	676	I	AAF34799	40S ribosomal protein S16	2E-66	M+M
ku2005-41	760	I	BAA13541	CPRD12 protein	6E-75	M+M
ku2005-56	204	I	AAA32857	receptor-like protein kinase	6E-5	E+M
ku2005-63	623	I	AAM62640	Function unknown protein	4E-22	M+M
ku2005-81	393	C	NP567449	acid phosphatase	3E-16	M+M
ku2005-100	534	C	ABA40465	ribosomal protein S27-like protein	5E-32	M+M
ku2005-118	656	I	AAS46243	xyloglucan endotransglucosylase -hydrolase, XTH7	7E-100	M+M
ku2500-154	156	C	CAD31840	alpha-dioxygenase	1E-6	E+M
ku2005-170	240	No	CAA48140	ubiquitin	4E-20	E+M
ku2005-175	461	I	NP197655	prephenate dehydratase	9E-16	M+M
ku2005-180	404	I	BAB33421	putative senescence-associated protein	2E-39	E+M
ku2005-181	619	I	AAB37746	expansin S1 precursor	7E-106	M+M
ku2005-232	522	C	NP197857	Function unknown protein	2E-46	M+M
ku2005-238	793	I	NP194345	transcription factor	6E-12	M+M
ku2005-243	468	C	NP178175	acetylornithine transaminase/ pyridoxal phosphate binding / transaminase	8E-31	M+M
ku2005-250	181	C	MU57845	disease resistance-responsive protein-related	9E-68	E+M
ku2005-267	116	I	NP850181	structural constituent of ribosome	8E-4	E+M
ku2005-272	148	I	MU62607	Function unknown protein	7E-31	E+M
ku2005-292	154	I	AAL25650	calcineurin-like protein	2E-19	E+M
ku2005-297	774	I	NP568239	UBP22 (UBIQUITIN-SPECIFIC PROTEASE 22)	1E-84	M+M
ku2005-314	284	C	AAT77309	Function unknown protein	3E-21	E+M
ku2005-321	127	C	NP200894	GTP-binding protein	9E-14	E+M
ku2005-336	318	C	NP197501	small GTP-binding nuclear protein; Ran-1	1E-17	M+M
ku2005-340	131	C	MU60193	ubiquitin-protein ligase	6E-65	E+M
ku2005-345	145	C	MU52543	Function unknown protein	8E-56	E+M
ku2005-350	412	C	XP482452	Putative protein kinase	4E-7	M+M
ku2005-363	265	I	NP193740	metallopeptidase	1E-17	E+M
ku2005-365	292	C	AAT77309	Function unknown protein	3E-23	E+M
ku2005-385	340	I	NP174119	carboxy-lyase	1E-28	E+M
ku2005-394	386	I	AAO24554	Function unknown protein	5E-38	M+M
ku2005-399	409	I	NP921921	Putative protein kinase	3E-17	M+M

Table S1. The putative functions (as determined by BLAST in GenBank) of the other transcript derived fragments (TDFs) responsive to the resistance of *C. metuliferus* infected with PRSV (continued)

TDF	Size (bp)	I/C[a]	Accession number	Functional annotation	BLAST *E*-value	Restriction site[b]
ku2005-409	735	I	MU54137	transferase	1E-150	M+M
ku2005-418	207	C	AAK93736	putative cysteinyl-tRNA synthetase	1E-12	M+M
ku2005-423	181	I	MU54308	60S ribosomal protein L13A (RPL13aD)	9E-28	E+M
ku2005-432	511	I	NP001030956	RPS15 (RIBOSOMAL PROTEIN S15)	2E-39	M+M
ku2005-440	207	I	1710320A	Photosystem I 20 kD protein	1E-22	E+M
ku2005-445	651	I	CAB72464	protein-tyrosine-phosphatase-like protein	2E-46	M+M
ku2005-449	592	I	XP450905	putative annexin	4E-63	M+M
ku2005-457	189	C	NP179688	Function unknown protein	5E-10	E+M
ku2005-462	659	C	BAD95352	bZIP-like protein	2E-20	M+M
ku2005-481	735	C	NP195540	transferase, transferring glycosyl groups	1E-32	M+M
ku2005-482	543	I	NP566542	Function unknown protein	2E-7	M+M
ku2005-483	349	I	NP172512	Function unknown protein	8E-39	M+M
ku2005-509	181	I	MU44891	CA1 (CARBONIC ANHYDRASE 1)	2E-72	E+M
ku2005-510	114	C	MU53888	carbonate dehydratase	4E-32	E+M
ku2005-512	228	I	MU43461	NtEIG-A1	7E-23	E+M
ku2005-515	176	I	MU62474	Function unknown protein	2E-16	E+M
ku2005-517	109	I	MU47871	bundle-sheath defective protein 2 family/bsd2 family	2E-36	E+M
ku2005-524	300	C	MU51610	Function unknown protein	1E-112	M+M
ku2005-529	480	I	NP918587	putative 33kDa oxygen evolvingprotein of photosystem II	4E-27	M+M
ku2005-544	224	I	P08474	Ribulose bisphosphate carboxylase small chain (RuBiSco)	2E-20	E+M
ku2005-554	11109	I	AAM73656	AER, auxin and ethylene cross-talk marker	1E-13	M+M
ku2005-561	872	C	AAD11808	syntaxin-related protein Nt-syr1	3E-57	M+M
ku2005-567	200	C	MU47813	ATBI1 (BAX INHIBITOR 1)	2E-75	E+M
ku2005-570	636	I	CAD87534	putative xyloglucan endotransglycosylase	2E-102	M+M
ku2005-571	1165	C	MU60959	AER	0	M+M
ku2005-573	991	C	NP196696	catalytic	9E-41	M+M
ku2005-574	677	C	NP177209	Kinase	3E-101	M+M
ku2005-577	863	C	MU43308	syntaxin-related protein Nt-syr1	0	M+M
ku2005-582	527	I	MU47524	thioredoxin h	0	M+M
ku2005-591	585	I	CAB61890	acetohydroxy acid isomeroreductase	8E-96	M+M

Table S1. The putative functions (as determined by BLAST in GenBank) of the other transcript derived fragments (TDFs) responsive to the resistance of *C. metuliferus* infected with PRSV (continued)

TDF	Size (bp)	I/C[a]	Accession number	Functional annotation	BLAST *E*-value	Restriction site[c]
ku2005-17	99	C		No significant similarity match		E+M
ku2005-27	130	I		No significant similarity match		E+M
ku2005-46	487	C		No significant similarity match		M+M
ku2005-51	386	I		No significant similarity match		M+M
ku2005-59	109	C		No significant similarity match		E+M
ku2005-86	483	C		No significant similarity match		M+M
ku2005-91	384	I		No significant similarity match		M+M
ku2005-105	172	I		No significant similarity match		E+M
ku2005-123	346	ND		No significant similarity match		M+M
ku2005-128	249	I		No significant similarity match		E+M
ku2005-137	251	C		No significant similarity match		E+M
ku2005-141	342	I		No significant similarity match		E+M
ku2005-149	136	C		No significant similarity match		E+M
ku2005-189	448	C		No significant similarity match		M+M
ku2005-227	479	C		No significant similarity match		M+M
ku2005-252	133	I		No significant similarity match		E+M
ku2005-262	111	I		No significant similarity match		E+M
ku2005-317	170	I		No significant similarity match		E+M
ku2005-380	430	I		No significant similarity match		M+M
ku2005-404	196	I		No significant similarity match		E+M
ku2005-417	303	C		No significant similarity match		E+M
ku2005-427	722	I		No significant similarity match		M+M
ku2005-435	678	C		No significant similarity match		M+M
ku2005-500	439	C		No significant similarity match		M+M
ku2005-519	93	C		No significant similarity match		E+M

[a] The transcription of TDFs is induced by virus infection (I) or constitutive (C) in *C. metuliferus*.
ND means non-detectable.

[b] The TDFs contain either restriction enzyme site of *EcoR* I or *Mse* I at the both ends.
